# Clinical, epidemiological, and laboratory prognostic factors in patients with leprosy reactions: A 10-year retrospective cohort study

**DOI:** 10.3389/fmed.2022.841030

**Published:** 2022-07-25

**Authors:** Douglas Eulálio Antunes, Diogo Fernandes Santos, Mayara Ingrid Sousa Lima, Larissa Pereira Caixeta, Meydson Benjamin Carvalho Correa, Emilly Caroline dos Santos Moraes, Natalia Carine Almeida Conceição, Luiz Ricardo Goulart, Isabela Maria Bernardes Goulart

**Affiliations:** ^1^National Reference Center for Sanitary Dermatology and Leprosy, Clinics Hospital, Federal University of Uberlândia, Uberlândia, MG, Brazil; ^2^Postgraduate Program in Health Sciences, School of Medicine, Federal University of Uberlândia, Uberlândia, MG, Brazil; ^3^Department of Biology, Federal University of Maranhão, São Luis, MA, Brazil; ^4^Institute of Biochemistry and Genetics, Federal University of Uberlândia, Uberlândia, MG, Brazil; ^5^Department of Medical Microbiology and Immunology, University of California, Davis, Davis, CA, United States

**Keywords:** antigens, leprosy, leprosy reaction, phenolic-glycolipid-1, survival analysis

## Abstract

**Introduction:**

Leprosy reactions, the main cause of neural damage, can occur up to 7 years after starting multidrug therapy. We aimed to approach the prognostic factors that may influence the leprosy reactions over the follow-up time.

**Methods:**

Retrospective cohort study, encompassing 10 years of data collection, composed of 390 patients, divided into 201 affected by reactions and 189 reaction-free individuals. Epidemiological, clinical, and laboratory variables were approached as prognostic factors associated with leprosy reactions. The association among variables was analyzed by a binomial test and survival curves were compared by the Kaplan-Meier and Cox proportional-hazards regression.

**Results:**

51.5% (201/390) of patients were affected by leprosy reactions. These immunological events were associated with lepromatous leprosy (16.2%; 63/390; *p* < 0.0001) and multibacillary group (43%; 169/390; *p* < 0.0001). This study showed that survival curves for the prognostic factor anti-PGL-I, comparing positive and negative cases at diagnosis, differed in relation to the follow-up time (Log Rank: *p* = 0.0760; Breslow: *p* = 0.0090; Tarone-Ware: *p* = 0.0110). The median survival times (time at which 50% of patients were affected by leprosy reactions) were 5 and 9 months for those reactional cases with negative (26/51) and positive serology (75/150), respectively. The time-dependent covariates in the cox proportional-hazards regression showed anti-PGL-I as the main prognostic factor to predict leprosy reactions (hazard ratio=1.91; *p* = 0.0110) throughout the follow-up time.

**Conclusions:**

Finally, these findings demonstrated that anti-PGL-I serology at diagnosis is the most important prognostic factor for leprosy reactions after starting multidrug therapy, thus enabling prediction of this immunological event.

## Introduction

Leprosy reactions, classified as Type 1 or Type 2, occur before, during and after Multi-Drug Therapy (MDT), and may be triggered by different co-infections and/or antigens of *Mycobacterium leprae* (*M. leprae*) especially phenolic-glycolipid-1 (PGL-I) (1, 2).

Regarding to the time for leprosy reaction occurrence, a study reported that 9.5% of patient had late type 1 reaction up to 7 years after starting MDT (3).

The type 1 reaction (T1R), common between borderline tuberculoid (BT), borderline borderline (BB), and borderline lepromatous (BL), might be subdivided into upgrading and downgrading reaction (4).

A current study showed 27% of patients being affected by T1R with 63% ranging from moderate to severe cases (5). Furthermore, a study reported that 60% of patients developed T1R with 90% of cases presenting inflamed plaques as the main sign (6). The T1R presents cutaneous manifestations such as erythema, infiltration into the skin and edema in preexisting lesions, as well as arising of new skin lesions. As to the presence of neurological signs and symptoms, it is possible to highlight neural thickening (edema), pain in the peripheral nerve, sensory-motor changes with loss of muscle strength and consequent evolution to physical disabilities (7).

The type 2 reaction (T2R), whose main presentation is erythema nodosum leprosum (ENL), is systemic and associated with the formation of immune complexes (IC) in the blood such as found in serum samples, and are deposited inside tissues, especially skin, kidneys and joints, reported as extravascular complements, therefore being a type III hypersensitivity reaction (8–10). This type of reaction is considered an immunological complication for the clinical forms BL and lepromatous leprosy (LL) (8). The T2R affected 44% of BL and 71% of LL according to a study involving a period of 12 years of data collection. Furthermore, independent on clinical form, it was reported 26.8% of T2R in a referral center (11, 12). The systemic signs and symptoms that are commonly present in this type of reaction include malaise, loss of weight and injury to internal organs, which in turn may cause peripheral neuropathy, orchiepididymitis, glomerulonephritis, myositis, arthralgia, iridocyclitis, hepatomegaly, and ganglion infarction (13). Hematological and biochemical changes may be present in T2R as leukocytosis, neutrophilia, thrombocytosis, increased acute-phase proteins such as C-reactive protein, alkaline phosphatase, transaminases, fibrinogen, and elevated immunoglobulins of the IgG and IgM classes (14).

Studies analyzing prognostic factors in relation to the outcome of leprosy reactions are scarce. However, it is possible to find some research studies limited to risk factors associated with leprosy reactions.

Therefore, we aim to approach, by means of comparison among survival curves, the prognostic factors that may be associated with leprosy reactions across 10 years of follow-up.

## Materials and methods

### Sample, place, and study design

Retrospective cohort study, involving a sample of 390 patients, divided into 201 affected by leprosy reactions and 189 reaction-free individuals, whose follow-up period ranged from 2006 to 2015. The secondary data were collected in the National Reference Center in Sanitary Dermatology and Leprosy at Federal University of Uberlândia, Brazil, from 2014 to 2016.

### Inclusion and exclusion criteria

The inclusion criteria were: leprosy patients affected by leprosy reactions type 1 and T2R; reaction-free patients, diagnosed by leprologists according to the clinical, histological and immunological criteria of Ridley and Jopling (15).

The exclusion criteria were: individuals with other chronic infectious diseases; patients affected by acute infections; relapses cases and/or patients with resistance to anti-leprosy drugs.

### Criteria for definition of leprosy reactions

The leprosy reactions (T1R and T2R) were identified and classified by the expert leprosy physician who evaluated the patient during the clinical episodes. The diagnosis was based on clinical and immunological criteria.

### Follow-up time

The follow-up time varied from time zero (t_0_) to time of event/outcome; the data collection encompassed a period of 10 years, as reported previously. In this present study, time zero (t_0_) was considered the date of the first dose of MDT to treat leprosy. On the other hand, the time-to-event/outcome was the first day of clinical manifestation of signs and symptoms associated with leprosy reactions. Each one of the patients have been followed for a total-person time of 7 years, by means of medical records, in order to registering the first leprosy reaction after starting the MDT.

### Clinical and epidemiological variables

The main clinical and epidemiological variables used in the study were: clinical form of the disease, type of leprosy reaction, operational classification (OC), period of leprosy reaction presentation, sex, age group, skin phenotype, and disability degree.

### Laboratory variables

The laboratory variables evaluated in this investigation were IgM antibodies to the PGL-I serology and dermal smear bacillary index.

Regarding anti-PGL-I serology, the cutoff point was equal to index 1. Thus, values below this point were negative and those above were positive. Indeterminate anti-PGL-I ELISA index values (equal to 1) were repeated. As to the bacillary index (BI) of dermal smear, the results equal to 0 were considered negative. On the contrary, BI values above 0 were classified as positive.

#### Anti PGL-I serology

The enzyme-linked immunosorbent assay (ELISA) was performed on all patients, against the native PGL-I molecule purified from the *M. leprae* cell wall, according to a methodology previously described in the literature (16).

#### Bacillary index of dermal smear

The mean of the dermal smear bacilloscopic index was obtained after collection of 7 standardized sites, such as: ear lobes, elbows, knees and main skin lesion. The BI, proposed by Ridley in 1962, is based on a logarithmic scale from 0 to 6, ranging from the absence of bacilli to the presence of more than 1,000 bacilli in each field examined (17).

## Ethical approval

This study was approved by the Research Ethics Committee at the Federal University of Uberlândia – Brazil under registration number 28931320.9.0000.5152. The written informed consent was not needed given that this research was to be carried out by means of secondary data.

### Statistical analysis

The binomial test was employed to compare the reaction and reaction-free groups regarding the proportions found in the epidemiological and clinical variables. Relative risk (RR) was used to assess the likelihood of the leprosy reactions in those individuals with the presence of factors assessed in this study. The comparison among survival curves was carried out by means of the Kaplan Meier test. The time-dependent covariates in the cox proportional-hazards regression was performed to ascertain the factors that influenced the outcome, leprosy reaction, in a multivariate model. The IBM Statistical Package for Social Sciences (SPSS) for Windows, version 22 (IBM Corp., Armonk, N.Y., USA) was used for all statistical analyses with a 5% significance level.

## Results

### Epidemiologic and clinical data

The sample was composed of 390 patients, 189 (48.5%) individuals were reaction-free and 201 (51.5%) affected by leprosy reactions. Among the reactive group, T1R predominated with 61.2% (123/201), while 38.8% (78/201) were T2R (Table 1). There was difference between the proportions of clinical form LL in the reactional individuals (31.3%; 63/201) when compared with the same clinical form in the reaction-free group (5.3%; 10/189) (*p* < 0.0001) (Table 1). All clinical and epidemiological variables are shown in Table 1.

**Table 1:** Comparison among proportions of Epidemiologic and clinical data from leprosy reaction and reaction-free groups by means of Binomial test.

		**Leprosy Reaction**		**Reaction-free**		**Total**		* **p**
		***n***	**%**	***n***	**%**	***n***	**%**	
Clinical form	I	0	0.0	4	2.1	4	1.0	
	T	8	4.0	49	25.9	57	14.6	<0.0001
	BT	58	28.9	98	51.9	156	40.0	<0.0001
	BB	37	18.4	12	6.3	49	12.6	0.0003
	BL	35	17.4	16	8.5	51	13.1	0.0088
	LL	63	31.3	10	5.3	73	18.7	<0.0001
		201		189				
Type of leprosy reaction	type 1	123	61.2					
	type 2	78	38.8					
Operational classification	PB	32	15.9	126	66.7	158	40.5	<0.0001
	MB	169	84.1	63	33.3	232	59.5	
Disabiity degree	0	102	50.7	142	75.1	244	62.6	<0.0001
	1	63	31.3	32	16.9	95	24.4	0.0009
	2	36	17.9	15	7.9	51	13.1	0.0035
Sex	Male	63	31.3	92	48.7	155	39.7	0.0005
	Female	138	68.7	97	51.3	235	60.3	
Skin phenotype								
	White	108	53.7	92	48.7	200	51.3	0.3183
	Brown	61	30.3	60	31.7	121	31.0	0.7655
	Black	16	8.0	18	9.5	34	8.7	0.5843
	Not declared	16	8.0	19	10.1	35	9.0	0.4699
Age group								
	0–19	3	1.5	13	6.9	16	4.1	0.0074
	20–39	45	22.4	43	22.8	88	22.6	0.9316
	40–59	105	52.2	87	46.0	192	49.2	0.2204
	≥60	48	23.9	46	24.3	94	24.1	0.9158

BB, borderline-borderline; BL, borderline-lepromatous; BT, borderline-tuberculoid; I, indeterminate; LL, lepromatous-lepromatous; MB, multibacillary; PB, paucibacillary; T, tuberculoid; T1R, type 1 reaction; ENL, erythema nodosum.

* Binomial test.

### Relative risk for the development of leprosy reactions

According to Table 2, the risk for leprosy reactions in those individuals with anti-PGL-I positive serology, at diagnosis, was 2.65 times more likely than in those with negative results (*p* < 0.0001; CI: 2.07–3.40). Table 2 highlights that the risk for leprosy reactions in individuals with positive dermal-smear BI at the diagnosis, was 2.56 times more likely than in those with negative results for the same test (*p* < 0.0001; CI: 2.05–3.20).

**Table 2:** Laboratory risk factors for leprosy reactions.

		**Leprosy Reaction**		**Reaction-free**		**Total**		**Relative Risk (RR)**		
		***n***	**%**	***n***	**%**	***n***	**%**	**RR**	***p***	**Confidence interval (CI)**
anti-PGL-I serology	Positive	150	74.6	55	29.1	205	52.6	2.65	<0.0001	2.07–3.40
	Negative	51	25.4	134	70.9	185	47.4			
Dermal smear bacillary index	Positive	139	69.2	43	22.8	182	46.7	2.56	<0.0001	2.05–3.20
	Negative	62	30.8	146	77.2	208	53.3			

### Survival curves, prognostic factors and time-to-event for leprosy reactions

**Figure 1:**
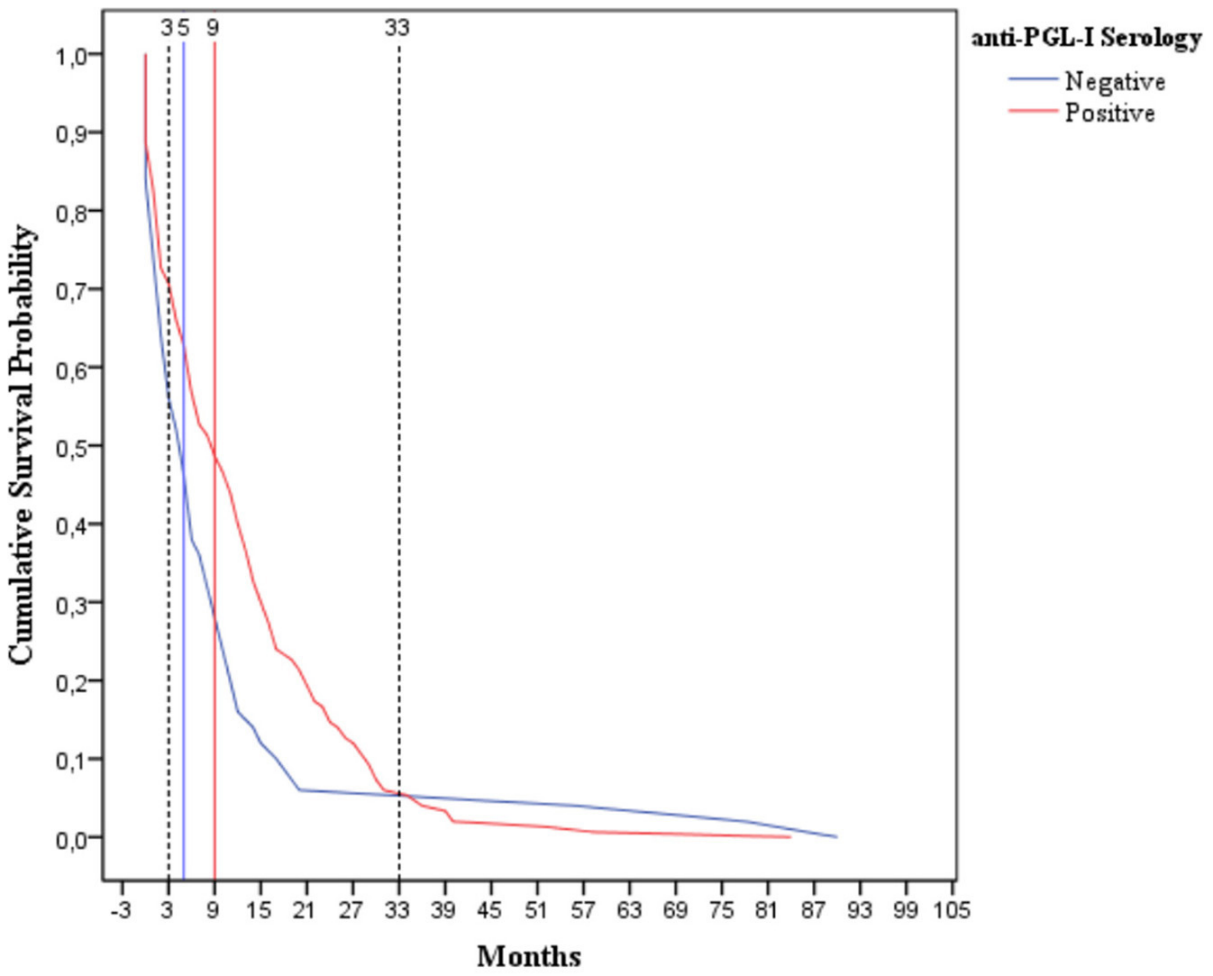
Survival curve (Kaplan-Meier) of 201 leprosy reaction individuals during the follow-up of 10 years according to the anti-PGL-I serology at diagnosis. The comparison between 2 cumulative survival probability curves that presented significant difference along all the time of follow-up (Log Rank, *p* = 0.076; Breslow, *p* = 0.009; Tarone-Ware, *p* = 0.011). Lines over all follow-up time: the blue line represents negative cases to anti-PGL1 serology (*n* = 51) and the red line the positive cases (*n* = 150).

The time-to-event, that is, from t_0_ to the first leprosy reaction (event/outcome), was determined by comparing survival curves assessing several prognostic factors that directly influenced the primary outcome, leprosy reaction. Figure 1 displays the comparison between two survival curves in those reactional individuals that were seronegative (*n* = 51) and seropositive (*n* = 150) for anti-PGL-I serology at diagnosis. It was observed that, within the first 3 months, 30% (45/150) of seropositive cases were affected by leprosy reactions, whereas 45% (23/51) seronegative ones presented this event within the same interval. The median survival times (times at which 50% of patients were affected by the event/leprosy reactions), were 5 and 9 months for those reactional cases with negative (26/51) and positive serology (75/150), respectively (Figure 1). Thus, reactional cases who presented negative serology had poor prognosis, due to the first reaction having occurred earlier after t_0_ when compared to seropositive cases. We emphasized that 33 months after the t_0_, the trend between prognostic factors changed, because the cases seronegative for anti PGL-I had better prognosis than seropositive patients. This finding may be confirmed by noting that after the 33rd month, the curve of seropositive individuals was under that of seronegative ones, indicating a higher leprosy reaction rate in seropositive patients after this period (Figure 1). Furthermore, the survival curves, as shown in Figure 1, were statistically different throughout the follow-up time cited in this study (Log Rank, *p* = 0.076; Breslow, *p* = 0.009; Tarone-Ware, *p* = 0.011).

**Figure 2:**
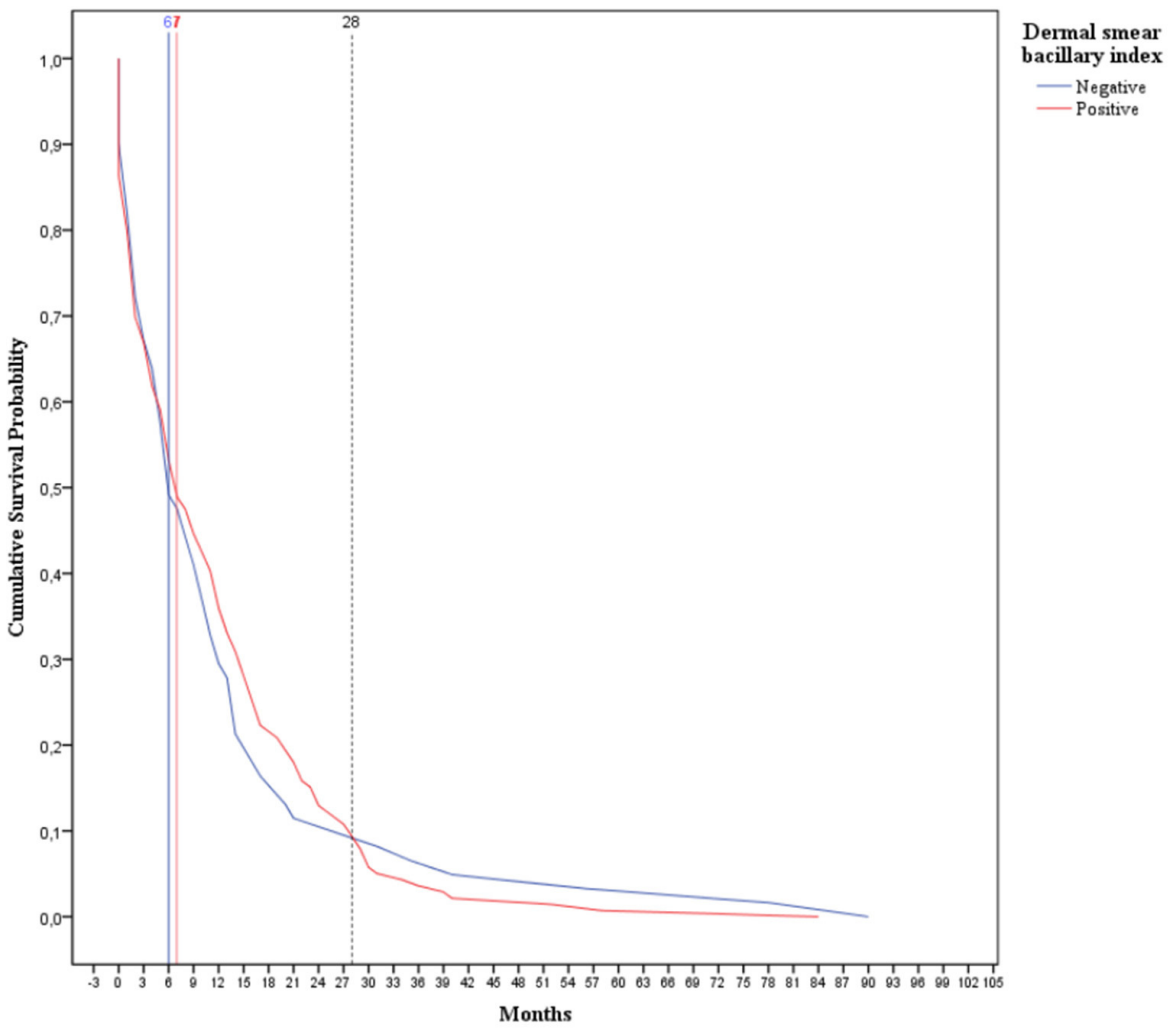
Survival curve (Kaplan-Meier) of 201 leprosy reaction individuals during the follow-up of 10 years according to dermal smear bacillary index at diagnosis divided into negative and positive. The comparison between 2 cumulative survival probability curves that presented significant difference along all the time of follow-up (Log Rank, *p* = 0.058; Breslow, *p* = 0.024; Tarone-Ware, *p* = 0.020).

As displayed in Figure 2, when was analyzed the BI of the dermal smear at diagnosis as a prognostic factor for leprosy reaction development, 50% (26/32) of those classified as negative at diagnosis presented reaction within 6 months after t_0_, whereas half of positive cases (85/169) had this same outcome at 7 months (Log Rank, *p* = 0.058; Breslow, *p* = 0.024; Tarone-Ware, *p* = 0.020).

With respect to sex, age group, degree of physical disability, clinical form and skin color, these factors did not show differences between the survival curves for the leprosy-reaction prognosis across the follow-up time (Supplementary Figures).

### Multivariate analysis of main prognostic factors

**Figure 3:**
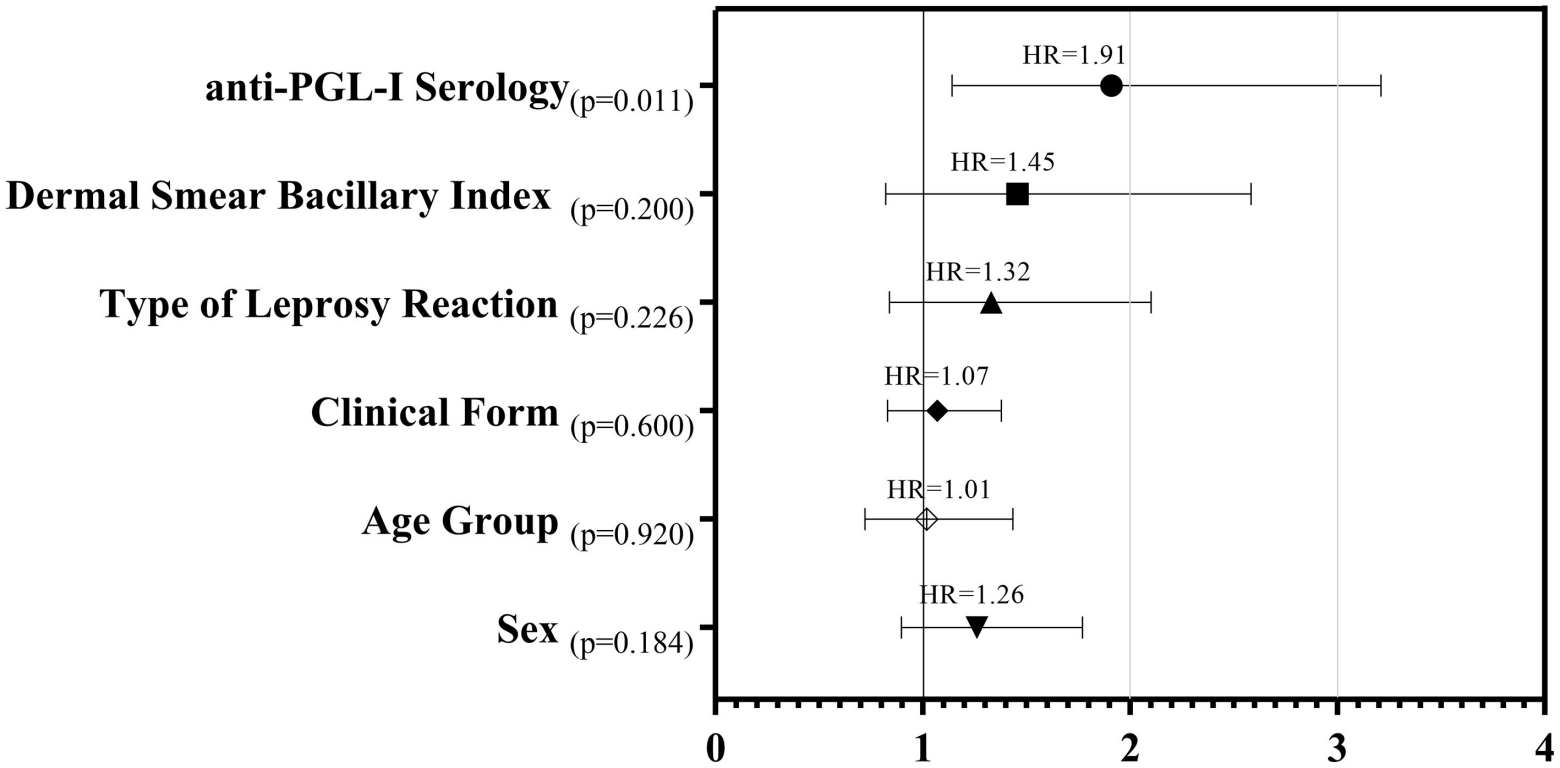
Forest plot of estimated *Hazard Ratios, p-values* and *confidence intervals* from epidemiologic, clinical and laboratory variables as prognostic factors for leprosy reactions - Cox Regression with time-dependent covariate analysis.

In Figure 3, a set of epidemiological (sex and age group), clinical (clinical form and type of leprosy reaction) and laboratory variables (anti-PGL-I serology and dermal smear Bacillary Index) were analyzed in a multivariate model, by means of the Cox Regression with time-dependent covariate analysis, in order to assess the more relevant prognostic factor. It was noted that anti-PGL-I serology was the principal prognostic factor with potential to predict the outcome, leprosy reaction, over the follow-up time with precision (*Hazard Ratio*: 1.91; *p* = 0.011) in a model with different factors.

### Summarized interpretation

By means of survival curves and Cox Regression with time-dependent covariate analysis, this study showed that the negative anti-PGLI at diagnosis may predict up to 33 months, an early reaction in this group (50% of leprosy reactions occurred until 5 months) with a proportional risk of nearly 2-fold (hazard ratio of 1.91) when compared with positive cases (50% of reactional cases occurred up to 9 months). On the contrary, after 33 months there is a poor prognostic for seropositive cases.

## Discussion

Among leprosy reaction cases, the borderline group, BT, BB and BL, have predominated, being considered by several studies as a risk factor for T1R (4, 5). The proportion of the reactional LL clinical form was higher than LL reaction-free in agreement with previous studies (13, 18). The low proportion of the reactional T form was expected, due to the fact that these cases may be subpolar tuberculoid (TTs), rare and immunologically unstable, being able to migrate on the clinical spectrum of disease toward borderline forms (19). The MB operational classification was associated with high bacillary load, which results in risk of leprosy reaction occurrence as reported in a past study (8). The disability degree 2, associated with leprosy reactions, corroborated a recently study that indicated a dependent relationship between these two variables (20). The association between female sex and leprosy reaction was cited in another study as a risk factor, especially for T1R (21). The low prevalence of reactional individuals that belong to the 0–19 age group may be related to the operational classification and clinical form of them, since they were treated in early stage of disease (22). Moreover, the efficient immune response in this group, since these individuals have regular production of B and T cells from bone marrow and the thymus (23). On the other hand, elderly individuals present an increased number of regulatory T lymphocytes (Treg), which may cause excessive suppression of immune responses; furthermore, degenerative disease associated with polypharmacy may favor immunologic abnormalities in the elderly (24).

The positivity of anti-PGL-I serology was mentioned in this study as a risk factor for leprosy reactions. Thus, this result may contribute as a risk factor for leprosy reaction when compared with those seronegative. This important finding is supported by a study that reported a high positivity proportion of this antigen in reactional individuals (25). Our results from dermal-smear BI indicated high risk for manifesting leprosy reaction when the results to this test were positive at diagnosis, as found in other studies that showed that positive BI raises the chance of developing leprosy reactions as compared with negative cases (7, 26).

The prognostic was poor among seronegative patients, due to half of seronegative individuals presented leprosy reaction up to 5 months, while those seropositive developed the event at 9 months after MDT (Figure 1). In this current research, the highest proportion of patients with negative anti-PGL-I serology at diagnosis, among reactional cases, belong to T and BT clinical forms (data not shown). These clinical forms exhibited T1R, cell-mediated immunity, with macrophage activation under the influence of cytokines such as tumor necrosis factor-α (TNF-α), interferon gamma (IFN-γ), IL-2 and Lymphotoxin-alpha (LT-α) (24). We hypothesized that early occurrence of this type of reaction in this seronegative to PGL-I IgM is associated with MDT action on *M. leprae* that after bacillus fragmentation releases antigens into the bloodstream activating Th1 response, predominant in these individuals (27). The humoral immune response prevailed in those cases with positive anti-PGL-I IgM serology, such as BL and LL, characterized by production of the cytokines L-4, IL-5 and IL-10, manifesting, therefore, T2R, which provokes an increase in circulating levels of TNF-α and IL-10 in some of them. It is important to highlight that IL-10 cytokine may favor bacillus survival and delay an efficient response against this mycobacterium (28, 29).

We emphasized in this present study that, 33 months after t_0_, there was a change in the prognostic-factor profile related to anti-PGL-I serology, evidencing poor prognosis for those seropositive, which may indicate a persistent of bacillary load in cases with higher dermal-smear BI. In relation to dermal-smear BI, a change in the prognostic factor was also observed 28 months after the t_0_. This previous finding might be related to the persistence of bacillary load, which is as common in MB as in BL and LL, which consequently, will present a time-dependent bacillary clearance (30). The bacillary clearance also depends on the immune competence of these clinical forms, given that, as reported previously, a longer duration was necessary to eliminate the bacilli from tissues in those with T2R when compared to those without T2R (30).

Leprosy patients classified as T and BT have developed reactions in less time when compared with BB and BL (Supplementary Table 1). This difference among clinical forms regarding the time-to-event may be associated with effective immune response against *M. leprae* in those individuals with low bacillary load, an immunological event, according to other authors, that occurs within 6 months (31). Half of LL individuals developed a leprosy reaction within 6 months after t_0_, corroborating a previous study that reported more than 70% of LL being affected by this reaction in the first 6 months after starting MDT treatment (32). The higher percentage of LL affected by T2R in the first 6 months after t_0_ is in accordance with risk factors associated with this clinical form, since BI > 4, and hypothetically related to activation of immune complexes and release of TNF-α by macrophages in these individuals with high BI (33).

The idea about the presence of immune complexes during T2R/ENL episodes may be reinforced by other research that assessed genic expression in peripheral blood mononuclear cell (PBMC) from T2R/ENL patients, demonstrating the high expression of components from the classical complement pathway, such as C1qA (34).

With respect to anti-PGL-I translational application, we recommend the use of this marker as prognostic factor in order to screening patients according to clinical forms and median time for the first leprosy reaction as shown in this research. This serology test may be suitable for creating an assistance flowchart involving esthesiometry, electroneuromyography, and medical assessment in a short period of time among evaluations to prevent nerve damage. The use of steroid such as prednisolone 20 mg/day during the first 4 months after MDT was pointed out in another research for leprosy reaction prevention. However, this strategy is controversial and more studies should be performed (35). The use of steroid associated with positivity of PGL-I after treatment with the goal to prevent leprosy reactions should be avoided, since the bacillary load of dermal-smear positive falls 1 log per year what may indicate the persistence of positivity of anti-PGL-I titers after treatment for multibacillary forms (36). Even though we did not focus on data after MDT, the positive anti PGL-I showed to be, in another study of our group, a predictive factor for peripheral nerve impairment demonstrated by electroneuromyography evidencing 4-fold chance of nerve damage for positive households contact as compared with seronegatives (17).

## Conclusion

Finally, this study showed that the anti-PGL-I should be considered the main prognostic factor for leprosy reactions prediction after MDT and pointed out a median time of 5 and 9 months for this event in seronegatives and seropositives, respectively. These data may facilitate the monitoring and follow-up of these patients in order to prevent potential peripheral neural damage.

The principal limitations of this study are related to the difficulty of testing cytokines and lipoarabinomannan (LAM) as prognostic factors for leprosy reactions in a large sample of patients, due to the high cost of these laboratory supplies.

## Data availability statement

The original contributions presented in the study are included in the article/Supplementary material, further inquiries can be directed to the corresponding authors.

## Ethics statement

The studies involving human participants were reviewed and approved by Research Ethics Committee at the Federal University of Uberlândia. Written informed consent for participation was not required for this study in accordance with the national legislation and the institutional requirements.

## Author contributions

DA, IG, LG, and DS designed the study. ML, LC, MC, EM, and NC collected data on reporting. The lab protocol was standardized and performed by ML, LC, MC, EM, and NC. DA performed the data analysis. DA and DS wrote the manuscript. Critical review was performed by IG and LG. IG and LG directed the research. All authors read and approved the manuscript.

## Funding

The authors thank the Brazilian funding agency CNPq for providing financial support to the National Institute of Science and Technology in Theranostics and Nanobiotechnology – INCT-TeraNano (CNPq-465669/2014-0).

## Conflict of interest

The authors declare that the research was conducted in the absence of any commercial or financial relationships that could be construed as a potential conflict of interest.

## Publisher's note

All claims expressed in this article are solely those of the authors and do not necessarily represent those of their affiliated organizations, or those of the publisher, the editors and the reviewers. Any product that may be evaluated in this article, or claim that may be made by its manufacturer, is not guaranteed or endorsed by the publisher.
