# Supplementary material for: Clinical, epidemiological, and laboratory prognostic factors in patients with leprosy reactions: A 10-year retrospective cohort study
Source: Front Med (Lausanne). 2022 Jul 25;9:841030. doi: 10.3389/fmed.2022.841030 (PMC9358030; doi:10.3389/fmed.2022.841030)
Supplement: Supplementary file 1 [file Data_Sheet_1.docx]

Supplementary Material

**Supplementary table 1.** Time-to-event analysis per clinical form, up to 3 months and greater than 3 months after the beginning of multidrug therapy

| Clinical form | **Time-to-event (leprosy reaction)** | | | |  |  |
| --- | --- | --- | --- | --- | --- | --- |
|  | **Up to 3 months** | | **Greater than 3 months** | |  |  |
|  | **n** | **%** | **n** | **%** | **total** | **%** |
| **T** | 7 | 87.5 | 1 | 12.5 | 8 | 4 |
| **BT** | 35 | 60.3 | 23 | 39.7 | 58 | 28.9 |
| **BB** | 12 | 32.4 | 25 | 67.6 | 37 | 18.4 |
| **BL** | 8 | 22.9 | 27 | 77.1 | 35 | 17.4 |
| **LL** | 22 | 34.9 | 41 | 65.1 | 63 | 31.3 |
|  |  |  |  |  | 201 | 100 |

**Legend**: BB: borderline-borderline; BL: borderline-lepromatous; BT: borderline-tuberculoid; I: indeterminate; LL: lepromatous-lepromatous; T: tuberculoid.

# Supplementary Figures


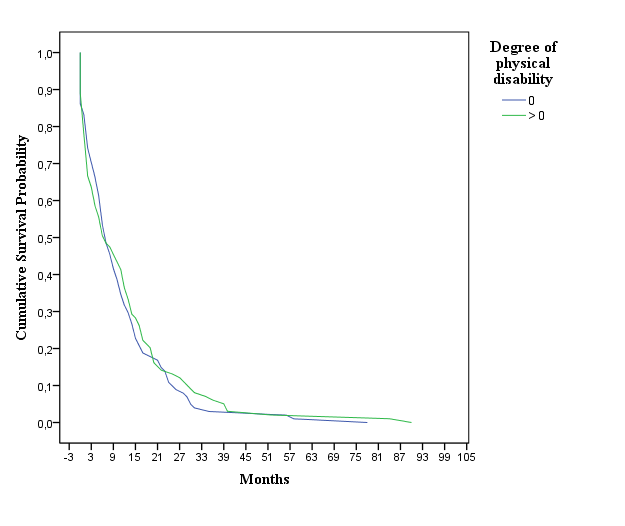


**Supplementary figure 1** - Survival curve (Kaplan-Meier) of 201 leprosy reaction individuals during the follow-up for 10 years according to the degree of physical disability. The comparison between 2 cumulative survival probability curves that presented no significant difference along all the time of follow-up, (Log Rank, p= 0.582; Breslow, p= 0.960; Tarone-Ware, p = 0.847).


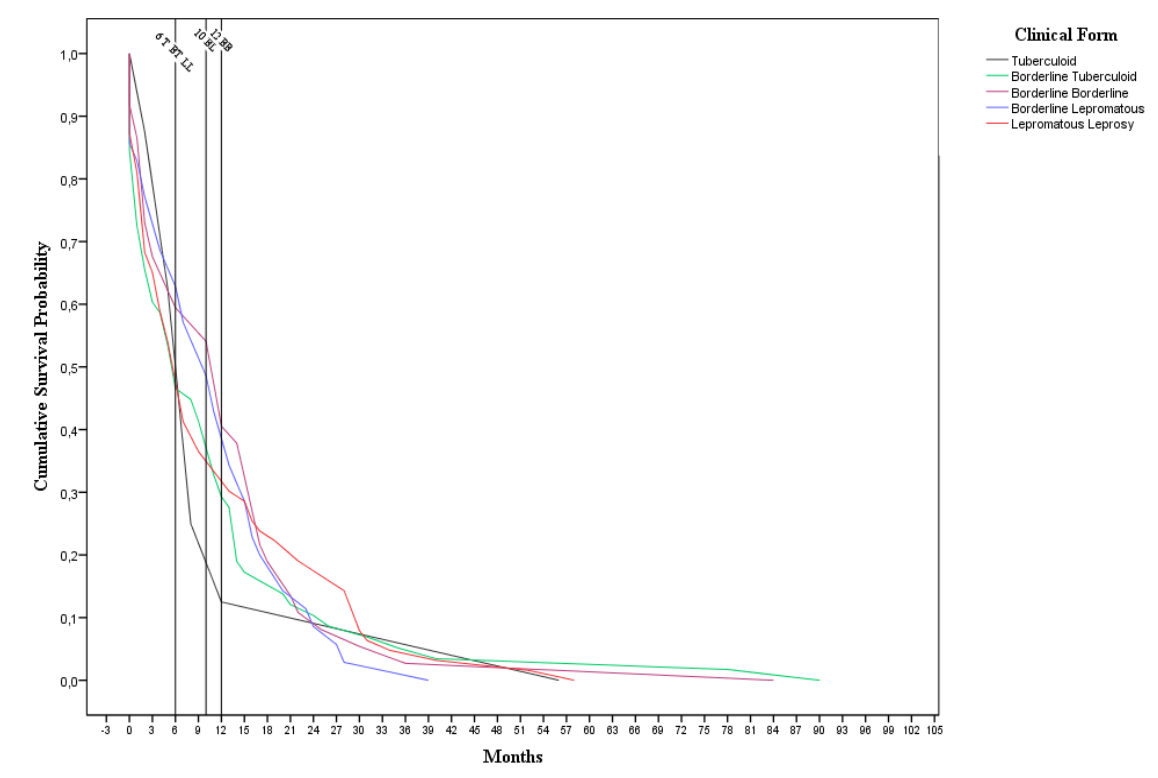


**Supplementary figure 2** **-** Survival curve (Kaplan-Meier) of 201 leprosy reaction individuals during the follow-up of 10 years according to the clinical form. The comparison among 6 cumulative survival probability curves presented no significant difference along all the time of follow-up, (Log Rank, p= 0.968; Breslow, p= 0.643; Tarone-Ware, p= 0.795). Lines over all follow-up time: the lines grey (tuberculoid; n=62), green (borderline tuberculoid; n=58), purple (borderline borderline; n=37), blue (borderline lepromatous; n=35) and red (lepromatous leprosy; n=63) represent the clinical forms of leprosy. Vertical black lines show median survival times at which the cumulative survival function is equal to 0.5 for tuberculoid (T), borderline tuberculoid (BT) and lepromatous leprosy (LL) (6 months); borderline lepromatous (BL) (10 months) and borderline borderline (BB) (12 months).


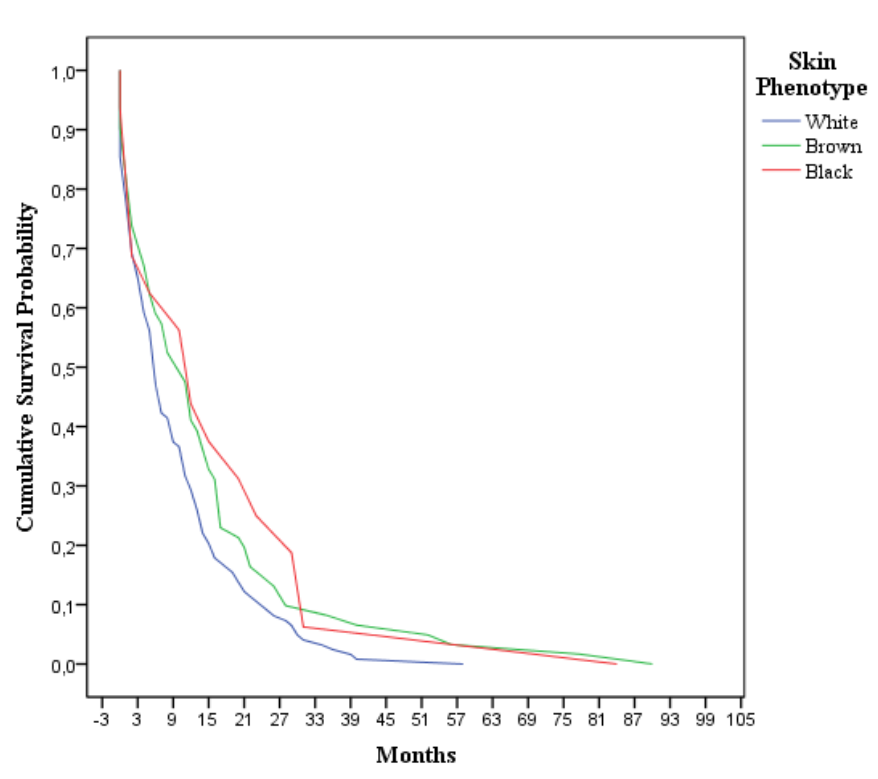


**Supplementary figure 3** - Survival curve (Kaplan-Meier) of 201 leprosy reaction individuals during the follow-up of 10 years according to the skin phenotype (White, Brown and Black). The comparison among 3 cumulative survival probability curves presented no significant difference along all the time of follow-up, (Log Rank, p= 0.042; Breslow, p= 0.118; Tarone-Ware, p= 0.074). Lines over all follow-up time: the blue line represents white (n=108), the green shows brown (n=61) and the red line the black cases (n=16).


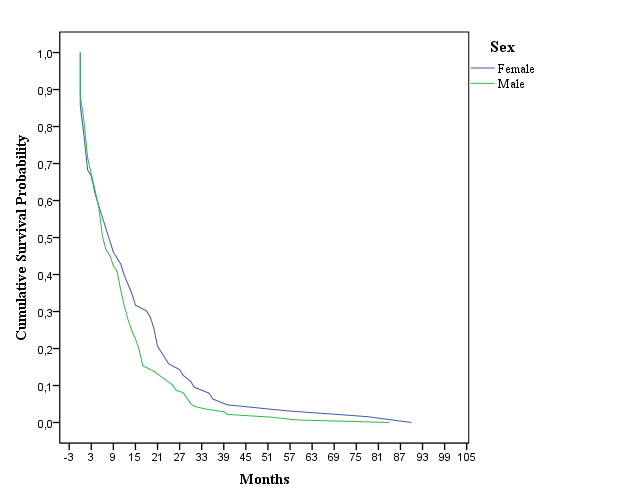


**Supplementary figure 4** - Survival curve (Kaplan-Meier) of 201 leprosy reaction individuals during the follow-up for 10 years according to the sex. The comparison between 2 cumulative survival probability curves that presented no significant difference along all the time of follow-up, (Log Rank, p= 0.148; Breslow, p= 0.483; Tarone-Ware, p = 0.285).


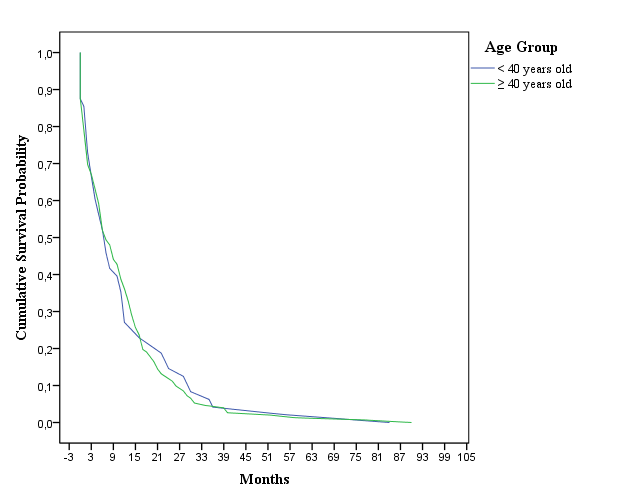


**Supplementary figure 5** - Survival curve (Kaplan-Meier) of 201 leprosy reaction individuals during the follow-up for 10 years according to the age group. The comparison between 2 cumulative survival probability curves that presented no significant difference along all the time of follow-up, (Log Rank, p= 0.816; Breslow, p= 0.997; Tarone-Ware, p = 0.935).
